# Polyunsaturated Fatty Acid and S-Adenosylmethionine Supplementation in Predementia Syndromes and Alzheimer's Disease: A Review

**DOI:** 10.1100/tsw.2009.48

**Published:** 2009-05-22

**Authors:** Francesco Panza, Vincenza Frisardi, Cristiano Capurso, Alessia D'Introno, Anna M. Colacicco, Alessandra Di Palo, Bruno P. Imbimbo, Gianluigi Vendemiale, Antonio Capurso, Vincenzo Solfrizzi

**Affiliations:** ^1^ Department of Geriatrics Center for Aging Brain, University of Bari, Bari, Italy; ^2^ Department of Geriatrics, University of Foggia, Foggia, Italy; ^3^ Research and Development Department, Chiesi Farmaceutici, Parma, Italy; ^4^ Internal Medicine Unit, IRCSS Casa Sollievo Dalla Sofferenza, Italy

**Keywords:** Alzheimer’s disease, mild cognitive impairment, S-adenosylmethionine, Sadenosylhomocysteine, n-3 polyunsaturated fatty acids

## Abstract

A growing body of evidence indicates that nutritional supplements can improve cognition; however, which supplements are effective remains controversial. In this review article, we focus on dietary supplementation suggested for predementia syndromes and Alzheimers disease (AD), with particular emphasis on S-adenosylmethionine (SAM) and polyunsaturated fatty acids (PUFA). Very recent findings confirmed that SAM can exert a direct effect on glutathione S-transferase (GST) activity. AD is accompanied by reduced GST activity, diminished SAM, and increased S-adenosylhomocysteine (SAH), the downstream metabolic product resulting from SAM-mediated transmethylation reactions, when deprived of folate. Therefore, these findings underscored the critical role of SAM in maintenance of neuronal health, suggesting a possible role of SAM as a neuroprotective dietary supplement for AD patients. In fact, very recent studies on early-stage AD patients and moderate- to late-stage AD patients were conducted with a nutriceutical supplementation that included SAM, with promising results. Given recent findings from randomized clinical trials (RCTs) in which n-3 PUFA supplementation was effective only in very mild AD subgroups or mild cognitive impairment (MCI), we suggest future intervention trials using measures of dietary supplementation (dietary n-3 PUFA and SAM plus B vitamin supplementation) to determine if such supplements will reduce the risk for cognitive decline in very mild AD and MCI. Therefore, key supplements are not necessarily working in isolation and the most profound impact, or in some cases the only impact, is noted very early in the course of AD, suggesting that nutriceutical supplements may bolster pharmacological approaches well past the window where supplements can work on their own. Recommendations regarding future research on the effects of SAM or n-3 PUFA supplementation on predementia syndromes and very mild AD include properly designed RCTs that are sufficiently powered and with an adequate length (e.g., 3–5 years of follow-up).

## INTRODUCTION

The burden of the age-related neurodegenerative diseases, particularly dementia, is expected to increase dramatically in both developed and developing nations[1]. Dementia is estimated to affect approximately 6% of the population aged 65 and older, with the prevalence increasing exponentially with age, being 40–70% at the age of 95 years and above[2]. In occidental countries, the most common forms of dementia are Alzheimer's disease (AD) and vascular dementia (VaD), with respective frequencies of 70 and 15% of all dementias[3]. The transitional phase between mild nondisabling cognitive decline and disabling dementia is an ambiguous diagnostic period during which it is unclear whether mild cognitive deficits predict incipient dementia or not. In the present article, we will use the term "predementia syndrome" to identify all conditions with age-related deficits in cognitive function reported in the literature, including a mild stage of cognitive impairment based on normal or pathological conditions considered predictive or early stages of dementia[4,5]. Such predementia syndromes have been defined for AD, but have not yet been operationalized for VaD and other specific forms of dementia.

In the last decade, advances in understanding the neurobiology of AD have translated into an increase in clinical trials that assess various potential AD treatments[6]. AD involves aberrant protein processing and is characterized by the presence of both intraneuronal protein clusters composed of paired helical filaments of hyperphosphorylated tau protein (neurofibrillary tangles [NFTs]) and extracellular protein aggregates (senile plaques [SPs]). Therefore, Alzheimer's classic pathological description of AD as a "two hallmarks disorder"[7] was confirmed by subsequent observations[8]. These neuropathological hallmarks of AD strongly influenced recent therapeutic approaches[9]. The SPs are the result of misprocessing of the amyloid precursor protein (APP), a type-1 transmembrane protein, by β- and γ-secretases to form a toxic β-amyloid (Aβ) peptide of 40–42 amino acids that aggregates and initiates a pathogenic self-perpetuating cascade, ultimately leading to neuronal loss and dementia[10]. Extracellular, and perhaps also intracellular, Aβ exert neurotoxic effects[11]. Extracellular Aβ peptides cluster in a β-sheet structure to form SPs. According to the "amyloid cascade hypothesis"[12], the development of SPs is thought to precede and precipitate the formation of NFTs as a result of the cellular changes invoked, and the oligomeric forms of Aβ peptide are the main cause of neuronal death in AD. APP may be metabolically processed according to two pathways. In the so-called nonamyloidogenic pathway, the α-secretase enzyme cleaves APP within the Aβ sequence and releases its transmembrane fragment sAPPα that appears to exert neuroprotective activity. In the amyloidogenic pathway, the β-secretase enzyme releases APP plus a 12-kDa protein fragment (C99), which in turn is cleaved by the γ-secretase enzyme giving way to Aβ. Accumulation of toxic aggregated forms of Aβ seems crucial in the pathogenesis of familial forms of AD[13], while many studies showed a weak correlation between Aβ deposits and cognitive status[14], and some showed that cognitively healthy elderly people could have substantial amyloid burden[15,16].

Indeed, the hypothesis that Aβ is the key pathologic factor affecting the disease process is strongly questioned by a recently published paper showing that although immunization with preaggregated $A\beta_{1-42}$ (AN1792) resulted in almost complete clearance of SPs from the brain of patients with AD, this plaque removal did not prevent progressive neurodegeneration[17]. Aβ may have a physiological role in modulating synaptic plasticity[18] and hippocampal neurogenesis[19]. Aβ deposition could simply represent a host response to an upstream pathophysiologic process[14] or serve a protective function[20], likely as an antioxidant/metal chelator[21,22]. Nevertheless, the fight against Aβ is continuing with more effective compounds.

A growing body of evidence indicates that nutritional supplements can improve cognition[23]; however, which supplements are effective remains controversial. At present, epidemiological studies have not suggested that intake of antioxidant vitamins is beneficial against dementia or AD[24,25]. Studies of supplementation with folic acid and B vitamins have yielded conflicting results[23,26,27]. In particular, in a multicenter, randomized, double-blind controlled clinical trial of high-dose folate, vitamin B6, and vitamin B12 supplementation (5 mg/day of folate, 25 mg/day of vitamin B6, 1 mg/day of vitamin B12) in 409 individuals with mild to moderate AD, although the vitamin supplement regimen was effective in reducing homocysteine levels, it had no beneficial effect on the primary cognitive measure, rate of change in Alzheimer's Disease Assessment Scale (ADAS)-cog score during 18 months, or on any secondary measures[28].

Some improvement in cognition has been achieved with other vitamins, such as vitamin E[29] and dietary fatty acids and fish oil, but also has been subject to controversy[30,31]. In fact, despite the wide use of vitamin E in the treatment of AD, this antioxidant compound was not effective in a prevention trial for mild cognitive impairment (MCI) to reduce progression to AD[32] nor clearly effective in patients with AD[33,34].

A very recent study, directly analyzing the levels of lipid peroxidation products from a large series of patients, found that although there are increases in the levels of oxidation products from patients with AD and other disease conditions, there was not the expected decrease in response to vitamin/antioxidant supplementation[35]. Sonnen and colleagues suggested that vitamin E intake in these patients was either taken at insufficient levels to alter oxidant balance or that other broader spectrum antioxidants may be required[35]. An alternative view is expressed in our questioning of the concept of oxidative stress as a simple equilibrium between oxidants and antioxidants. Instead, this concept may be interpreted as a finely tuned and robust system that regulates the oxidant balance that primarily depends on metabolic reducing power, and the complex interplay between endogenous (glutathione) and exogenous (vitamins) reductants[36]. Beyond a deficiency in antioxidant vitamins, their excess is also detrimental[36], possibly because the pro-oxidant/antioxidant balance is a complex self-correcting system that regulates the system to a set point equilibria necessary to maintain physiology. This concept has been recently confirmed by a study in which 55 AD patients were recruited and divided into two groups: placebo or treated with 800 IU of vitamin E per day for 6 months. In the first group of patients, "respondents" to vitamin E, blood oxidized glutathione (GSSG) levels were lower after the treatment and scores on the cognitive tests were maintained. The second group, "nonrespondents", consisted of patients in which vitamin E was not effective in preventing oxidative stress. In these patients, cognition decreased sharply to levels even lower than those of patients taking placebo. Based on these findings, it appears that vitamin E lowers oxidative stress in some AD patients and maintains cognitive status; however, in those in which vitamin E does not prevent oxidative stress, it is detrimental in terms of cognition. Therefore, supplementation of AD patients with vitamin E cannot be recommended without determination of its antioxidant effect in each patient[37].

Since 2002, the PREADVISE (Prevention of AD by Vitamin E and Selenium) trial has recruited patients, with an expected final enrollment of 10,400 people (estimated study completion date: December 2012), and only participants who are taking part in the SELECT study (a study that looks at the use of vitamin E and selenium for preventing prostate cancer) may apply to participate in the PREADVISE study[38]. In addition to vitamin E, several antioxidants have been suggested for the modulation of brain metal metabolism and attenuation of oxidative stress for the prevention and treatment of AD[39]. Finally, the National Institute on Aging (NIA) has very recently ended enrollment for a Phase IB clinical trial to measure the effects of vitamin E, ascorbic acid (vitamin C), thioctic acid (α-lipoic acid), and coenzyme-Q on markers of oxidative stress in AD[40]. Furthermore, the epidemiological evidence of an association between a reduced risk of AD and a diet high in polyunsaturated fatty acids (PUFA)[41] is further supported by recent findings that certain diets have been associated with a lower incidence of predementia syndromes[30,42,43,44], i.e., MCI or age-related cognitive decline (ARCD).

Correct assessment of the efficacy of supplementation is further compromised by any underlying dietary deficiency[45]. A larger question is whether supplements can boost cognition under healthy conditions or offset cognitive loss. These are not the same result and supplements that are effective in one instance may not be effective in the other. In fact, Ginkgo biloba, which has long been considered to improve cognitive performance, was recently shown not to delay the onset of AD[46,47]. Not all supplements that maintain health should also be expected to prevent disease-related, or perhaps even age-related, decline[48,49]. Studies in animal and cell culture models indicate that antioxidant protection derived from nutritional supplementation with key foods or individual agents is associated with improved cognition[50,51,52,53,54]. In this review article, we focus on dietary supplementation suggested for predementia syndromes and AD, with particular emphasis on S-adenosylmethionine (SAM) and PUFA.

## S-ADENOSYLMETHIONINE IN ALZHEIMER'S DISEASE

As seen above, antioxidants such as vitamin E provide some, but not complete, neuroprotection in AD[55,56]. Limitations of vitamin E are likely to be due, at least in part, to its lipophilic nature and resultant inability to quench cytosolic oxidative species, including those resulting from antecedent membrane oxidation[57]. An additional approach may be to stimulate the production of endogenous antioxidants. The endogenous antioxidant glutathione (GSH) and activity of the associated glutathione-S-transferase (GST; EC 2.5.1.18) enzymes are reduced in AD[58,59]. Thus, the role of GST isoenzymes as risk factors for AD could be important; in particular, GSTs detoxify commonly encountered products generated by oxidative damage, and reduced GST activity has been reported in multiple brain regions and in ventricular cerebrospinal fluid in short postmortem-interval AD patients[59]. Oxidative stress activates the GSTs, with the M1, T1, and P1 variants acting to detoxify numerous products of oxidizing reactions that cause damage to nucleic acids, lipids, and proteins[60]. An association between late-onset AD and genetic variants of GSTP1 (V allele) and GSTT1 (0/0 genotype) was also recently found[60], confirming other findings also suggesting that polymorphisms of this enzyme with diminished activity potentiate the impact of apolipoprotein E (APOE) deficiency[61]. Strategies to maintain appropriate GSH production may be useful as part of a therapeutic approach to delay the onset or progression of AD. GSH itself cannot be taken up; however, the GSH precursor N-acetyl cysteine (NAC) increases GSH production and demonstrated some efficacy in clinical trials[62]. Folate deficiency contributes to many neurological and psychiatric disorders including AD[63]. Folate- and B12-dependent reactions regenerate methionine from the neurotoxin homocysteine, which is related to the severity and progression of AD. The deleterious effects of folate deprivation are potentiated by deficiency in APOE, which itself increases oxidative stress and is associated with AD[64]. Functional folate deficiency can also arise from polymorphisms in 50,100 methylene tetrahydrofolate reductase (MTHFR, the enzyme that uses folate), which represent synergistic AD risk factors along with APOE deficiency[65]. Supplementation with folate and/or B12 in AD has generated conflicting results[66]. Folate deficiency decreases SAM, the major methyl donor, which declines in normal aging and AD, and may underlie the gradual hypomethylation of DNA that accompanies aging[67]. APOE deficiency also fosters a critical reduction in SAM and because SAM is an essential cofactor for GST, restricts the ability of GSH to quench cytosolic oxidative species[68]. Diminished SAM in AD may foster increased expression of presenilin (PS), leading to an increase in Aβ, the pathological hallmark of AD, and β- and γ-secretase activity, the enzymes responsible for the abnormal cleavage of the APP[69,70]. Although SAM provided limited efficacy in clinical trials for depression[71], its effect in AD remains unknown.

A very recent study by Tchantchou and colleagues demonstrated that SAM increased GST activity in murine brain homogenates. In particular, this study observed that SAM increased GST activity in homogenates of APOE–/– mice (transgenic mice lacking in APOE, a model for age-related oxidative damage) in a dose-response manner[53], which were devoid of SAM and demonstrated reduced GST activity, but not normal mice, which had normal SAM and GST activity *in situ*[65]. These results were consistent with the ability of dietary supplementation with SAM to restore normal levels of GST activity in APOE−/− mice maintained on the deficient diet, but not to increase GST activity in normal mice maintained on the complete diet. As seen above, decreased GSH represents an early event in neurodegeneration[72]. GSH peroxide (GPX) and GSH reductase (GR) display increased activity in AD, yet GST undergoes a paradoxical decrease in activity, despite increased oxidative damage[59,73]; the reason for this imbalance in GSH enzymes remains unclear. Prior studies demonstrated that SAM supplementation improves working memory and can compensate for cognitive decline, depression, and increased aggression, including that accompanying dietary folate deficiency and deficiency in APOE[50,74]. Similar to the situation in AD[59,73], brain tissue of APOE−/− mice displayed reduced levels of SAM, and underwent an even further reduction in SAM and an increase in S-adenosylhomocysteine (SAH), the downstream metabolic product resulting from SAM-mediated transmethylation reactions when deprived of folate[65].

From this research area, a very recent case-control study determined the plasma concentrations of SAH, SAM, and homocysteine, and the erythrocyte composition of phosphatidylcholine (PC), phosphatidylethanolamine (PE), and their respective PUFA concentrations in 26 patients with AD and 29 healthy control subjects[75,76]. In this report, there was a significant increase in the plasma concentrations of SAH and homocysteine, and a significant increase in the plasma concentrations of SAM in the AD patients. There was a significant decrease in the erythrocyte content of PC and an increase in the erythrocyte content of PE in the AD patients. The erythrocyte PC from AD patients had a significant depletion of docosahexaenoic acid (DHA), an n-3 PUFA, and arachidonic acid (ARA), an n-6 PUFA[75,76]. There was a significant negative correlation between plasma SAH and the DHA composition of erythrocyte PC, reflecting the inhibition of hepatic phosphatidylethanolamine N-methyltransferase (PEMT) activity by SAH in AD. The PEMT pathway also plays an important role in the mobilization of ARA into plasma[77]. The inhibition of hepatic PEMT by SAH may influence the production of ARA-derived regulatory lipids, such as prostaglandins. Several investigators have found inverse associations between objective measures of cognitive function and plasma or serum homocysteine concentrations in patients with AD, suggesting that homocysteine can serve as a predictor of cognitive performance[63,78]. The most common cause of hyperhomocysteinemia is considered to be a deficiency of folate or vitamin B12[79]. In fact, although the catabolic rate of homocysteine results from the interaction between genetic makeup and B vitamin status, it is generally accepted that elevated plasma homocysteine concentrations are a sensitive marker for folate and vitamin B12 tissue deficiency[80,81]. It has been shown that plasma homocysteine is a better correlate of cognitive function than the serum folate or vitamin B12 concentrations themselves[81], thus indicating a model for the relationship between subclinical vitamin deficiency and cognitive function[81].

However, very recently, an open-label pilot study on early-stage AD patients and a placebo-controlled study on moderate- to late-stage AD patients were conducted on a nutriceutical supplementation that included SAM[82,83] (see Table). In fact, in these two studies, the nutriceutical formulation investigated consisted of folic acid (400 mg), vitamin B12 (6 mg), vitamin E (30 IU), SAM (400 mg), NAC (600 mg), and acetyl-L-carnitine (ALCAR) (500 mg). Nutriceutical formulation was prepared at United States Pharmacopeia (USP) grade under Food and Drug Administration (FDA)–approved, cyclic guanosine monophosphate (cGMP) conditions in tablet form, with two tablets constituting a daily dose, by Nutricap Labs (Farmingdale, NY). In the first study, at baseline and at 3-month intervals for a total of 12 months, 14 mild to moderate AD patients completed the protocol. Participants were offered the opportunity to continue with the nutriceutical formulation beyond the 12-month trial with a requirement for caregiver reports only. A subset (N = 7) of these caregivers supplied information for their family members at 18 months[82]. Participants improved in the Dementia Rating Scale 2 (DRS-2) and the Clock Drawing Tests (Clox 1 and 2) (CDT) (neuropsychological performance). Family caregivers reported improvement in multiple domains of the 12-item Neuropsychiatric Inventory (NPI) (abnormal behavior affecting the patient's well-being) and maintenance of performance in the Alzheimer's Disease Cooperative Study–Activities of Daily Living (ADCS-ADL) (ability to engage in day-to-day activities). Sustained performance was reported by caregivers for those participants who continued in an 11-month

**TABLE**
**Principal Clinical Trials on PUFA and SAM Supplementation in Patients with MCI, VaD, AD, and ARCD**

| Participants | Interventions and Duration of Exposure | Outcome Measures | Effects of Interventions | Ref. |
|---|---|---|---|---|
| 20 Elderly nursing home residents with VaD | A single dose of 4.3 g of DHA was administered; dose effect was not assessed. The duration of exposure was 12 months. | Cognitive functioning was evaluated using HDS-R and MMSE scores at baseline, and after 3, 6, and 12 months. | Baseline HDS-R and MMSE scores were 15 to 22, consistent with mild to moderate dementia. HDS-R and MMSE scores improved in the DHA-treated group, but not among patients who were not treated with DHA. Comparisons between groups were significant at 3 and 6 months for the HDS-R and at 6 months for the MMSE. | [95] |
| 204 Patients with mild to moderate AD and with acetylcholine esterase inhibitor treatment and a MMSE > 15 points. | A single dose of 1.7 g of DHA plus 0.6 g of EPA was administered. The duration of exposure was 6 months placebo-controlled and 6 months open for both groups. | Primary outcome measures: MMSE and ADAS-cog. Secondary outcome measures: global function as assessed with the CDR. | Administration of n-3 PUFA in patients with mild to moderate AD did not delay the rate of cognitive decline according to the MMSE or ADAS-cog. However, positive effects were observed in a small group of patients with very mild AD (MMSE > 27 points). | [96] |
| 21 Patients with mild cognitive dysfunction (12 MCI patients with supplementation and nine MCI patients with placebo), 10 patients with organic brain lesions, and eight patients with AD. | A single dose of 240 mg/day of ARA and DHA, or 240 mg/day of olive oil (placebo). The duration of exposure was 3 months. | The cognitive functions were evaluated using the Japanese version of RBANS at two time points: before and 90 days after the supplementation. | The MCI group with supplementation showed a significant improvement of the immediate memory and attention score. The organic group showed a significant improvement of immediate and delayed memory. However, there were no significant improvements of each score in AD and MCI placebo groups. | [101] |
| 204 Patients with mild to moderate AD and with acetylcholine esterase inhibitor treatment and a MMSE > 15 points. | A single dose of 1.7 g of DHA plus 0.6 g of EPA was administered. The duration of exposure was 6 months placebo-controlled and 6 months open for both groups. | Neuropsychiatric symptoms were measured with NPI and MADRS. Caregivers' burden and activities of daily living (DAD) were also assessed. | No significant overall treatment effects on neuropsychiatric symptoms, on activities of daily living, or on caregivers' burden were found. However, significant positive treatment effects on the scores in the NPI agitation domain in APOE ε4 carriers and in MADRS scores in non-APOE ε4 carriers were found. | [97] |
| 23 Patients with mild to moderate AD and 23 patients with MCI. | n-3 PUFA 1.8 g/day in monotherapy or placebo (olive oil). The duration of exposure was 24 weeks. | Global clinical function measured with CIBIC-plus, cognitive function with ADAS-cog and MMSE, depressive symptoms with HDRS. | This supplementation may improve global clinical function (CIBIC-plus) in MCI patients relative to placebo. No associations were found between randomization group and ADAS-cog, MMSE, or HDRS scores. | [102] |

*Table continues*

**TABLE (continued)**

| Participants | Interventions and Duration of Exposure | Outcome Measures | Effects of Interventions | Ref. |
|---|---|---|---|---|
| 14 Community-dwelling individuals with early-stage AD. | Vitamin/nutriceutical formulation (folate, vitamin B6, vitamin E, SAM, NAC, and ALCAR) in an open-label trial. The duration of exposure was 12 months. | At baseline and at 3-month intervals for a total of 12 months, participants completed the DRS-2 and the CDT ; caregivers completed the ADCS-ADL, and the 12-item NPI. | Participants improved in the DRS-2 and CDT. Caregivers reported improvement in multiple domains of the NPI and maintenance of performance in the ADCS-ADL. Sustained performance was reported by caregivers for those participants who continued in an 11-month extension. | [82] |
| Independently living individuals (n = 302) aged ≥ 65 years; CES-D score < 16, MMSE score > 21. | A single dose of 1,800 mg/day EPA+DHA (n = 96), 400 mg/day EPA+DHA (n = 100), or placebo capsules (n = 106). The duration of exposure was 26 weeks. | Changes in mental well-being were assessed as the primary outcome with the CES-D, MADRS, GDS-15, and HADS-A. | Treatment with neither 1800 nor 400 mg EPA+DHA differentially affected any of the measures of mental well-being after 13 or 26 weeks of intervention compared with placebo. | [31] |
| Independently living individuals (n = 302) aged ≥ 65 years; CES-D score < 16, MMSE score > 21. | A single dose of 1,800 mg/day EPA+DHA (n = 96), 400 mg/day EPA+DHA (n = 100), or placebo capsules (n = 106). The duration of exposure was 26 weeks. | Cognitive performance was assessed using an extensive neuropsychological test battery that included the cognitive domains of attention (SC-WT; fWDST), sensorimotor speed (TMT-A), memory (WLT; bWDST), and executive function (TMT-B; VFT). | There were no significant differential changes in any of the cognitive domains for either low- or high-dose fish oil supplementation compared with placebo; an effect of EPA-DHA supplementation in subjects who carried the APOE ε4 allele was also found, but only on the cognitive domain of attention. | [108] |
| 12 Institutionalized patients diagnosed with moderate- to later-stage AD. | Vitamin/nutriceutical formulation (folate, vitamin B6, vitamin E, SAM, NAC, and ALCAR) randomly assigned to treatment group. The duration of exposure was 9 months. | At baseline and at 3-month intervals for a total of 9 months, participants completed the DRS-2 and the CDT; caregivers completed the ADCS-ADL and the 12-item NPI. | Participants receiving the formulation demonstrated a clinically significant delay in decline in the DRS-2 and the CDT as compared to those receiving placebo. Institutional caregivers reported approximately 30% improvement in the NPI and maintenance of performance in the ADCS-ADL or more than 9 months. | [83] |

bWDST = Backward Test of the Wechsler Digit Span Task; CDR = Clinical Dementia Rating Scale; CES-D = Center for Epidemiologic Studies Depression Scale; CIBIC-plus = Clinician's Interview-Based Impression of Change Scale; DAD = Disability Assessment for Dementia; EPA = Eicosapentaenoic Acid; fWDST = Forward Test of the Wechsler Digit Span Task; GDS-15 = 15-Item Geriatric Depression Scale; HADS-A = Hospital Anxiety and Depression Scale; HDRS = Hamilton Depression Rating Scale; HDS-R = Hasegawa's Dementia Rating Scale; MADRS = Montgomery Asberg Depression Scale; MMSE: Mini-Mental State Examination; RBANS = Repeatable Battery for Assessment of Neuropsychological Status; SC-WT = Stroop Color–Word Test; TMT-A = Trail Making Test Version A; TMT-B = Trail Making Test Version B; VFT = Verbal Fluency Test; WLT = Word Learning Test.

extension[82] (Table). Although the present study suffers from the lack of a placebo, nutriceutical formulation efficacy over the course of 12 months exceeded that of historical placebos from three studies on mild to moderate AD[84,85,86]. Furthermore, in a small cohort of 12 institutionalized patients diagnosed with moderate- to later-stage AD, participants were randomly separated into treatment or placebo groups. Participants receiving the nutriceutical formulation demonstrated a clinically significant delay in decline in the DRS-2 and Clox 1 and 2 as compared to those receiving placebo[83] (Table). Institutional caregivers reported approximately 30% improvement in the NPI and maintenance of performance in the ADCS-ADL for more than 9 months[83].

In particular, the beneficial effect on irritability and agitation/aggression in the NPI is likely to be derived principally from SAM, which reduced aggression in transgenic mice harboring the APOE ε4[82]; the underlying biochemical mechanism is not clear, but may relate to restoration/improved neurotransmitter balance, as SAM restored acetylcholine levels in mice, including that resulting from folate deficiency[50]. These promising findings provided direct support that nutritional intervention can have a positive effect on progression of early-stage AD and even at advanced stages. They further suggested that a combinatorial approach can provide superior neuroprotection more than individual supplements and suggest that this nutriceutical formulation may potentiate pharmacological approaches. The small participant populations in both of these studies suggested that the efficacy of nutriceutical formulation be interpreted with caution and that larger placebo-controlled trials are warranted.

## POLYUNSATURATED FATTY ACIDS IN PREDEMENTIA SYNDROMES AND ALZHEIMER'S DISEASE

Recently, a number of dietary elements and foods have been reported to be either risk or protective factors for the development of dementia and AD. These include fat, fatty acids, antioxidants, fish, homocysteine/methionine, vitamins, and alcohol[87], suggesting that the connection among diet, dementia, and aging may be oxidative stress with a possible central role for dietary antioxidants[88]. The epidemiological evidence of an association between a reduced risk of AD and a diet high in PUFA, particularly DHA[38,41], is further supported by recent findings that certain diets have been associated with a lower incidence of predementia syndromes[43,89]. In fact, findings from the Italian Longitudinal Study on Aging (ILSA) demonstrated that high monounsaturated fatty acids (MUFA), PUFA, and total energy intake were significantly associated with a better cognitive performance in ARCD subjects in a 8.5-year follow-up[43]. Furthermore, findings from the same population-based study demonstrated that while dietary fatty acid intakes were not associated with incident MCI, high PUFA intake appeared to have a borderline nonsignificant trend for a protective effect against the development of MCI[83,89]. Moreover, plasma levels of n-3 PUFA appear to be directly associated with cognitive function and are found to be lower in AD patients[90,91]. Also, phospholipid fatty acid profiles in the brains of AD patients are altered[86,92]. In contrast, high consumption of fish, an important source of n-3 PUFA, has been associated with a reduced risk of AD development[44,93].

One randomized clinical trial (RCT), using an n-3/n-6 fatty acid compound for 4 weeks for 100 AD patients (60 received the fatty acid compound and 40 a placebo control), found improvements in mood, cooperation, appetite, sleep, ability to navigate in the home, and short-term memory[94]. Furthermore, another RCT assessed the effect of supplementation with DHA on cognitive function among 20 elderly nursing home residents with VaD. Cognitive functioning was evaluated using the Hasegawa's Dementia Rating Scale (HDS-R) and Mini-Mental State Examination (MMSE) scores at baseline and after 3, 6, and 12 months. Baseline HDS-R and MMSE scores were 15 to 22, consistent with mild to moderate dementia. HDS-R and MMSE scores improved in the DHA-treated group, but not among patients who were not treated with DHA. Comparisons between groups were significant at 3 and 6 months for the HDS-R and at 6 months for the MMSE[95] (Table).

Recently, Freund-Levi and colleagues examined the effects of dietary n-3 PUFA supplementation, randomizing 204 patients with moderate AD to receive DHA and eicosapentaenoic acid (EPA) (for a total dose of 1,720 mg DHA/600 mg EPA) or placebo for 6 months (OmegAD Study). After the treatment period, all of the subjects received open-label n-3 PUFA for another 6 months. The authors found that the supplementation did not delay the rate of cognitive decline but, in the group of 32 patients with the most mild AD (MMSE > 27, Clinical Dementia Rating Score 0.5–1), n-3 PUFA supplementation slowed the decline in MMSE scores[96]. In addition, the subjects in the placebo group of these very mild AD patients also showed a statistically significant slowing of decline when they were switched to treatment between 6 and 12 months, suggesting that n-3 PUFA might be of benefit to slow the progression of the disease in MCI or very mild AD[96] (Table). Furthermore, this supplementation did not result in marked effects on neuropsychiatric symptoms in mild to moderate AD patients except for possible positive effects on depressive symptoms and agitation symptoms in subgroups[97]. In fact, there were positive effects on depressive symptoms in non-APOE ε4 carriers and in APOE ε4 carriers on agitation symptoms[97] (Table). Furthermore, very recent findings from the OmegAD Study suggested that a DHA-enriched n-3 PUFA supplement may positively affect weight and appetite in patients with mild to moderate AD. Not carrying the APOE ε4 allele and high DHA were independently associated with weight gain[98]. Finally, in the OmegAD project, the 6-month treatment with a DHA-rich n-3 PUFA preparation was associated with clear effects on released cytokines from peripheral blood mononuclear cells stimulated *ex vivo* with lipopolysaccharide. A significant decline of released interleukin (IL)-1β, IL-6, and granulocyte colony-stimulating factor (G-CSF) was found, whereas tumor necrosis factor-α (TNF–α), IL-8, -10, and granulocyte-macrophage CSF secretions were not significantly lower at 6 months. However, the G-CSF response was not significantly different statistically compared with the response of the placebo group[99]. The clinical significance of the cytokines and growth factors analyzed in the OmegAD project is further emphasized by the recent report by Ray and colleagues that showed that plasma levels of IL-1, TNF, and G-CSF are strong predictors of development of AD[100].

Furthermore, the effect of ARA and DHA after a 90-day supplementation on MCI, organic brain lesions, or AD showed a significant improvement of the immediate memory and attention score for MCI patients, and a significant improvement of immediate and delayed memories for patients with organic brain damage[101] (Table). The AD group showed no improvement after the supplementation of ARA and DHA, and the placebo group showed no significant improvement of cognitive functions by the supplementation of 240 mg/day of olive oil (high MUFA content)[101] (Table). Finally, the preliminary results from a 24-week, randomized, double-blind placebo-controlled study on 23 participants with mild or moderate AD and 23 with MCI randomized to receive n-3 PUFA 1.8 g/day or placebo (olive oil), suggested that n-3 PUFA monotherapy was well tolerable for most of the participants with AD or MCI[102] (Table). This supplementation may improve global clinical function, as measured by the Clinician's Interview-Based Impression of Change (CIBIC) Scale, which included caregiver-supplied information (CIBIC-plus), relative to placebo. No associations were found between randomization group and ADAS-cog, MMSE or Hamilton Depression Rating Scale (HDRS) scores. Levels of EPA on erythrocyte membrane were associated with cognitive function, measured by ADAS-cog, in these patients[102]. However, in a secondary analysis, participants with MCI showed more improvement on the ADAS-cog than those with AD associated with n-3 PUFA administration[102] (Table), which supports recent reports that indicate that PUFA supplementation could be more effective on cognition in people with very mild AD[96] or MCI[101].

There is a correlation between the fatty acid composition of the brain and that of circulating erythrocytes[103]. The decreased concentrations of DHA in n-3 PUFA deficiency can be reversed by the administration of a DHA diet[101], but the inhibition of PEMT by SAH could still inhibit the uptake of DHA into lipoproteins and impair its transport to peripheral tissues[77]. The most effective means for lowering plasma homocysteine is B vitamin supplementation (a combination of folate, vitamin B12, and vitamin B6)[63]. Some authors suggested that the possible metabolic link between the increased production of SAH and phospholipid metabolism, with a decreased mobilization of DHA from the liver into plasma and peripheral tissues, may increase the risk of atherosclerosis and stroke leading to cerebrovascular and neurodegenerative changes in AD[75]. The use of a combination of n-3 PUFA, folic acid, and vitamin B12 may be a more effective means of increasing the uptake of DHA into the brain than a diet high in n-3 PUFA alone. In fact, elevations in plasma levels of homocysteine is an emerging risk factor for AD, but it remains an open question whether interventions designed to lower plasma homocysteine concentrations will improve cognitive function or retard the rate of cognitive decline in older adults with or without AD[63]. Homocysteine is a sulfur-containing amino acid that can induce apoptosis and cause increased neuronal vulnerability to excitotoxicity by mechanisms mainly involving DNA damage[104]. Formed through methionine metabolic conversion, homocysteine is metabolized by remethylation and trans-sulfuration pathways. The remethylation pathway is controlled by vitamin B12–dependent methionine synthase and methylenetetrahydrofolate reductase, whereas vitamin B6–dependent cystathionine β synthase takes part in the trans-sulfuration pathway. Reduced activity of any of these three enzymes would result in increased homocysteine plasma levels[105]. Furthermore, the accumulation of alleles of single-nucleotide polymorphisms that decrease the activity of the two main enzymes involved in homocysteine degradation (i.e., methionine synthase and cystathionine β synthase) was found to augment the risk of developing AD[106], supporting the hypothesis that homocysteine metabolism genetics is directly involved in AD pathogenesis. It would be interesting to consider whether an increasing uptake of PUFA into the brain will decrease the risk for AD and cognitive decline as well. The very recent findings of the study by Tchantchou and colleagues confirmed that SAM can exert a direct effect on GST activity[53]. Since AD is accompanied by reduced GST activity, diminished SAM, and increased SAH, these findings underscored the critical role of SAM in maintenance of neuronal health, suggesting a possible role of SAM as a neuroprotective dietary supplement in AD.

A few years ago, a Cochrane review concluded that there was a growing body of evidence from biological, observational, and epidemiological studies that suggested a protective effect of n-3 PUFA against dementia. However, the Cochrane review team was unable to locate a single published RCT on which to base recommendations for the use of dietary or supplemental n-3 PUFA for the prevention of cognitive impairment or dementia[107]. However, very recently, in a randomized, double-blind, placebo-controlled trial of 302 cognitively healthy (MMSE score > 21) individuals aged 65 years or older, the possible impact of n-3 PUFA on the mental well-being and cognitive performance of nondepressed (CES-D score < 16) older individuals was investigated[31,108] (Table). In this RCT, participants were randomly assigned to 1,800 mg/day EPA-DHA, 400 mg/day EPA-DHA, or placebo capsules for 26 weeks[31,108]. In older Dutch subjects, no effect of daily supplementation with low or high doses of EPA-DHA on mental well-being as assessed by depression and anxiety questionnaires was found[31] (Table). Furthermore, there were no significant differential changes in any of the cognitive domains (attention, sensorimotor speed, memory, and executive function) for either low- or high-dose fish oil supplementation compared with placebo[99,108]. However, an effect of EPA-DHA supplementation in subjects who carried the APOE-ε4 allele was found, but only on the cognitive domain of attention[99,108] (Table). An improvement in men after 26 weeks of intervention for the low-dose fish oil group compared with placebo was also found, although these subgroup analyses did not include adjustment for multiple comparisons. Fish oil may be beneficial in these subjects who are most sensitive to developing dementia. These two substantially negative studies on ARCD may be explained by the samples investigated (nondepressed and noncognitively impaired older subjects). Further trials in depressed patients or APOE ε4 carriers with MCI are needed. Finally, there is another ongoing RCT with cognitive endpoints of n-3 PUFA supplementation in healthy cognitively intact older persons. The Older People And n-3 Long-chain polyunsaturated fatty acid (OPAL) study is a double-blind, randomized, placebo-controlled trial examining the effect of daily supplementation with 700 mg n-3 PUFA (500 mg DHA and 200 mg EPA) for 24 months on cognitive performance in healthy older persons aged 70–79 with good cognitive function (MMSE ≥ 24 out of 30 points at baseline) who are recruited from 20 primary care practices[109]. The OPAL study was completed at the end of 2007 and findings will be published shortly. At present, only baseline data are available, suggesting once again that higher fish consumption is associated with better cognitive function in later life[110].

The mechanisms by which high unsaturated fatty acid (UFA: MUFA or PUFA) intake could be protective against cognitive decline and dementia in healthy older people are, at present, unknown. In the older subjects of the ILSA, which fulfilled a Mediterranean dietary pattern, total fat is 29% of energy, with a high consumption of olive oil (46 g/day), a MUFA energy intake of 17.6% of total energy, 85% of which derived from olive oil, and a saturated fatty acid (SFA) intake of only 6%[111]. In this population, the prolonged protection of MUFA intake against age-related changes in cognitive functions may be linked to the relevant quota of antioxidant compounds in olive oil, including low molecular weight phenols[112]. In fact, animal studies suggested that diets high in antioxidant-rich foods, such as spinach, strawberries, and blueberries, rich in anthocyanins and other flavonoids may be beneficial in slowing age-related cognitive decline[113]. The possible role of antioxidant compounds from olive oil do not diminish or otherwise alter the argument concerning the fatty acids because this is only a possible explanation of the role of MUFA on age-related cognitive changes in our population, in which MUFA intake derived for a large part from olive oil.

The protective effect of dietary UFA could be related to the role of fatty acids in maintaining the structural integrity of neuronal membranes, determining the fluidity of synaptosomal membranes and thereby regulating neuronal transmission. Furthermore, essential fatty acids can modify the activity of certain membrane-bound enzymes (phospholipase A2, protein kinase C, and acetyltranferase), and the function of the neurotransmitters' receptors. Finally, free fatty acids, lipid metabolites, and phospholipids modify the function of membrane proteins, including ion channels[114]. Moreover, fatty acid composition of neuronal membranes in advancing age demonstrated an increase in MUFA content and a decrease in PUFA content[115]. There is also evidence associating a dietary deficiency of n-3 PUFA with changes in cortical dopaminergic function[116]. The n-3 PUFA from fish may be inversely associated with dementia because it lowers the risk of thrombosis[117], stroke[118], cardiovascular disease[119], and cardiac arrhythmia, reducing the risk of thromboembolism in the brain and consequently of lacunar and large infarcts that can lead to VaD and AD. Furthermore, the n-3 PUFA may be important as lipids in the brain, particularly for the possible influence of DHA on the physical properties of the brain that are essential for its function[120]. Furthermore, fish oil was a better source than α-linoleic acid for the incorporation of n-3 PUFA into rat brain phospholipid subclasses[30]. On the contrary, high linoleic acid intake (n-6 PUFA) may increase the susceptibility of LDL cholesterol to oxidation, which makes it more atherogenic[121], even if the association between linoleic acid and atherosclerosis is controversial[122]. Therefore the ratio of dietary n-3/n-6 PUFA intake may influence the potential role of PUFA on cognitive decline and dementia, the optimal ratio of n-6:n-3 for an healthier diet should be <5:1[123]. Finally, a high dietary intake of SFA and cholesterol increases the risk for cardiovascular disease, and therefore for cognitive decline, VaD, and AD[124]. On the contrary, treatment for 4 weeks with a Mediterranean-inspired diet rich in n-3 PUFA decreased blood lipids in healthy individuals with a low-risk profile for cardiovascular disease, with a beneficial effect also on vascular function and oxidative stress[125].

## CONCLUSIONS

Recent findings suggested that SAM can exert a direct effect on GST activity, and AD is accompanied by reduced GST activity, diminished SAM, and increased SAH, the downstream metabolic product resulting from SAM-mediated transmethylation reactions when deprived of folate. Therefore, several experimental findings underscored the critical role of SAM in maintenance of neuronal health, suggesting a possible role of SAM as a neuroprotective dietary supplement in AD[126]. Very recently, a nutriceutical formulation consisting of six vitamins and nutriceuticals (folic acid, vitamin B12, vitamin E, ALCAR, NAC, and SAM) showed improved cognitive performance in AD. However, to be effective, this supplementation should be started prior to extensive cognitive decline; this nutriceutical formulation improved cognitive performance for more than 2 years when treatment was initiated during the early stages of AD, but was only capable of delaying the decline vs. placebo when initiated later in the course of the disease[82,83]. In subjects who already have severe cognitive impairment or dementia, it might be too late for dietary supplementation to counteract the process of cognitive decline. Furthermore, epidemiological evidence suggested a possible association between PUFA (particularly, n-3 PUFA) and reduced risk of dementia. However, due to the small number of studies that discuss this topic, further research is necessary before a strong conclusion can be drawn. Some recent RCTs assessed the cognitive or functional effect of n-3 PUFA supplementation on patients with VaD, AD, MCI, or ARCD in cognitively unimpaired older subjects. These RCTs suggested a positive effect of this intervention only in very mild AD or MCI patients, or in subgroups (e.g., APOE ε4 carriers), for cognitive performance in nondemented subjects, or for neuropsychiatric symptoms in mild to moderate AD patients.

In conclusion, for mild AD and predementia syndromes, we suggest a high-risk condition for progression to dementia of vascular and degenerative origin: intervention trials using measures of dietary supplementation (dietary n-3 PUFA and SAM plus B vitamin supplementation) to determine if such supplements will reduce the risk for cognitive decline. Therefore, key supplements are not necessarily working in isolation, or in solely the "predicted" or anticipated manner, and the most profound impact, or in some cases the only impact, is noted very early in the course of AD. This is to be expected in that serious damage to neurons may exert a protracted loss, but one that cannot be reversed by nutrition alone. This does bring up the thought, however, that nutriceutical supplements may bolster pharmacological approaches well past the window where supplements can work on their own. Recommendations regarding future research on the effects of SAM or n-3 PUFA supplementation on predementia syndromes and very mild AD include properly designed RCTs that are sufficiently powered and with an adequate length (e.g., 3–5 years of follow-up). In particular, the future RCTs on PUFA supplementation should address the effects of different types of n-3 PUFA (i.e., DHA, EPA, ARA, and total n-3 PUFA) as well as the ratio of n-6 to n-3 PUFA. Finally, these RCTs should be designed to include a baseline assessment of dietary n-3 and n-6 PUFA intake, and to evaluate the effect of dose, treatment duration, and the sustainment of effect after discontinuation of n-3 PUFA consumption.
